# Prediction of Stress–Strain Curves Based on Hydric Non-Destructive Tests on Sandstones

**DOI:** 10.3390/ma12203366

**Published:** 2019-10-15

**Authors:** Marco Ludovico-Marques, Carlos Chastre

**Affiliations:** 1 Barreiro School of Technology, Polytechnic Institute of Setúbal, Barreiro, 2839-001 Lavradio, Portugal; ludovicomarques@gmail.com; 2 CERIS and Department of Civil Engineering, FCT, Universidade Nova de Lisboa, 2829-516 Caparica, Portugal

**Keywords:** building stone, reservoir sandstone, NDT, water absorption, compression, analytical model

## Abstract

The study of the mechanical behavior of building stones is traditionally supported by destructive compression tests carried out on representative specimens. However, in order to respect the monuments’ integrity, the study of the mechanical behavior of stones can be based mostly on physical properties obtained from non-destructive tests (NDT). For this study, a simple and cheap NDT—water absorption under low pressure—was used to carry out fast surveys and to predict the most important design parameters of loadbearing masonry, among which are the compressive strength, strain at failure, and even elastic modulus on monument blocks. The paper presents the results of the experimental work conducted to obtain the physical properties and stress–strain curves of the sandstones tested. Supported by these results, it was possible to correlate the various parameters and develop an analytical model that predicts the stress–strain curve of the sandstones based on water absorption under low pressure tests. A good agreement is observed between the analytical model and the experimental tests.

## 1. Introduction

Sandstones are widely distributed on the world’s cultural built heritage, being dominant in central Europe and also with a relevant presence in Southern Europe, the Middle East, and North Africa, as well as in East Asia. [1]. Besides that, sandstones are outstanding in rock-art cave sites and in open-air archaeological rock-art sites in North America, the Middle East, North Africa, East Asia, and Australia. The sandstone rock masses, encompassing sandstones, siltstones, and conglomerates, are the most representative reservoirs for petroleum and gas rock mass in the world (circa 75%) [2]. The economic importance of rock masses for sandstone in the oil and gas industry is an additional motivation to apply the testing and modeling methodology herein presented to rock samples in order to contribute to an early phase in the exploration of fields. In Portugal, the use of sandstones is common in the cultural heritage near the Atlantic Ocean, especially in the West (Alcobaça, Peniche, and Lourinhã) and South (Silves). In these ancient buildings, sandstone dimension stones are generally in massive masonry walls, allowing the characterization of the mechanical behavior of these buildings essentially as loadbearing masonry. Suitable conservation and rehabilitation of cultural built heritage should be based on adequate techniques applicable on materials and structures, following the main principles of rehabilitation, i.e., understanding of the existing materials, ensuring their physical integrity, maintenance, and reversibility (International charters of Athens cited in Ref. [3], Venice [3], and Krakow [4]). The importance of proper diagnosis is fundamental in cultural built heritage (ICOMOS, 2004) [5], and it should be noted that the removal of material for physical and mechanical characterization is against the quasi non-invasive rehabilitation principle. As a consequence, alternative non-destructive techniques to assess the physical and mechanical behavior of building stones are strongly recommended by the scientific community. Foraboschi [6] revealed the importance of structural design on the development of outstanding monument constructions.

The compression mechanical parameters depend on the rocks’ physical parameters, as referenced by the following authors: Goodman (1989); Palchick (1999); Hatzor and Palchick (1997); Tuğrul and Zarif (1999); Palchick and Hatzor (2002); Palchick and Hatzor (2004), Vásárhelyi and Ván (2006); Yilmaz (2010); Ludovico-Marques and Chastre et al. (2012); Heidari et al. (2017a, 2017b); Chastre and Ludovico-Marques (2018) [7,8,9,10,11,12,13,14,15,16,17,18]. In general, the decrease of compressive and tensile strength is associated with the increase of porosity and water absorption under low pressure behavior related to the increment of pores, voids, and microcracks. The weathering effect on the physical and mechanical properties of rocks were studied by Tan et al. [19] and Noor-E-Khuda et al. [20], in which freeze-thaw cycles were studied, and by Foraboschi and Vanin [21], Ludovico-Marques and Chastre [22], who focused on the salt effect on bricks and on sandstones, respectively. 

**Figure 1:**
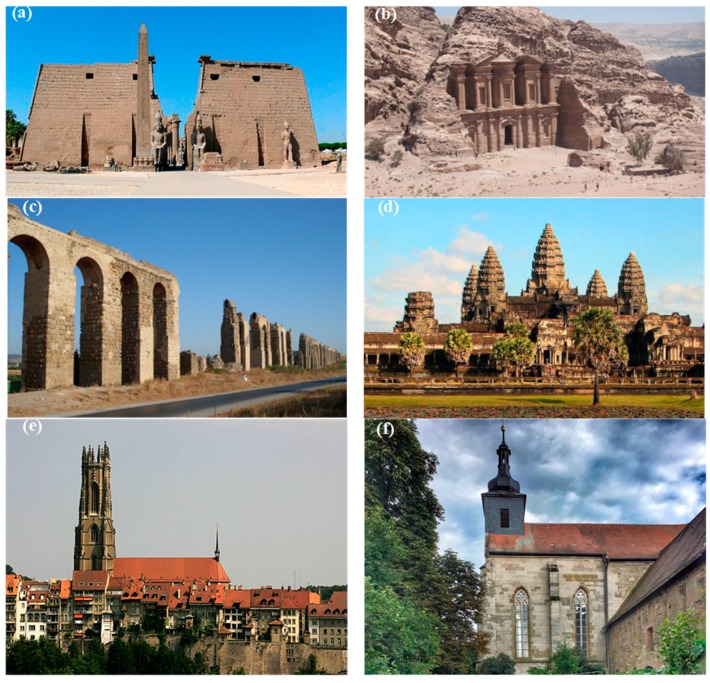

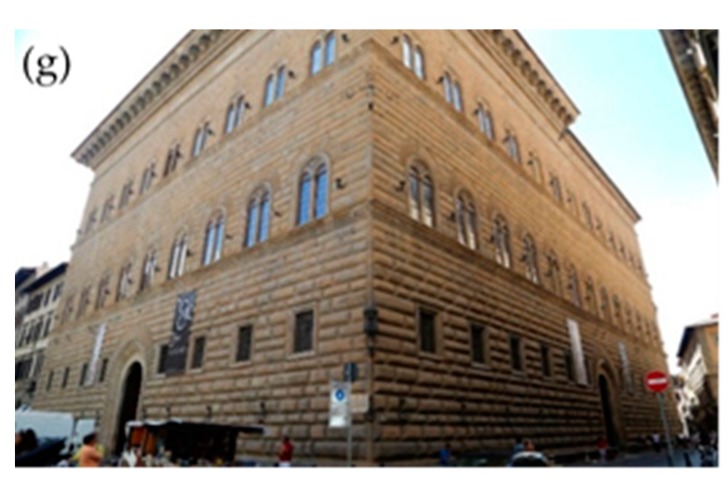
Sandstone monuments in the world. (**a**) Luxor temple in Tebas (Upper Egypt); (**b**) El Deir (“The Monastery”) in Petra (Jordan); (**c**) Carthage aqueduct in Tunisia; (**d**) Angkor Wat in Cambodia; (**e**) St. Nicolas Cathedral in Switzerland; (**f**) Birkenfeld Monastery in Germany; (**g**) Strozzi Place in Florence (Italy).

Malesani and Vannucci [23], Félix [24], Sorace [25], Banchelli et al. [26], Uchida et al. [27], Tiano et al. [28]; Fitzner et al. [29], Zoghlami et al. [30], and Heinrichs [31] have studied some of the most important sandstone monuments in the world (Figure 1) and reported their mineralogical, physical, and mechanical properties, summarized in Table 1 and Table 2. 

**Figure 2:**
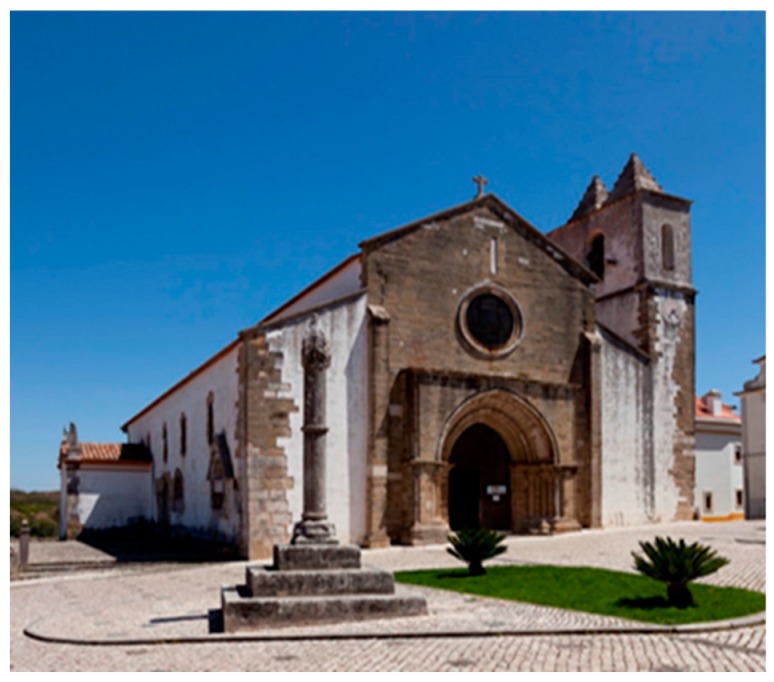
St. Leonard Church: View of the main façade (West) of the sandstone national monument in Atouguia da Baleia with its Gothic vault.

Figure 1, Table 1 and Table 2 report the main features of these important sandstone monuments in order to allow a proper comparison between them and the sandstone monument studied in this paper (Figure 2).

These building stones show a general mineralogical composition of circa 60% to 90% quartz, 0% to 15% feldspars, 0% to 25% clays, and some vestigial mica. The average grain size in the sandstones ranges from 0.08 to 0.4 mm and are generally medium to fine-grained. The analysis of the particle sizes revealed a uniform distribution. The porosity values range between 2% and 26%, with a higher value of 35% obtained on the Egyptian monuments referred in Table 1. The bulk density values vary between 1800 and 2400 kg/m^3^, and the real density values range between 2550 and 2670 kg/m^3^. These monuments’ building stones show values of ultrasonic pulse velocity of compressional waves (UPV) of about 1200 to 2200 m/s perpendicular to the bedding and between circa 1300 to 2600 m/s parallel to the bedding. The latter reaches 4000 m/s on Angkor sandstones.

Ludovico-Marques and Chastre reported that Ehrenberg and Nadeau [32] showed a statistical analysis of porosity and permeability values of siliciclastic reservoirs studied around the world. The results show average values of porosity of 13% encompassing 90% of these reservoirs and median values of porosity of circa 24%, obtained from depths between 0 and 250 m. Average values of permeability of 18 mD, reported in 90% of the reservoirs of these rock masses, and median values of permeability of 95 mD were given by the median statistical class defined by porosity values ranging between 17.5% and 22.5%. Jizba [33] shows the experimental data obtained on physical and mechanical tests carried out in the laboratory on sandstone cores collected in gas wells on Travis Peak formation in East Texas, from about 2000 m up to 3000 m. These sandstones are mostly quartz arenites and sub-litharenites according to Folk [34]. The clay content varies between 2% and circa 15%. The permeability values range between 0.13 and 25 mD. The unconfined uniaxial compressive strength of specimens (UCS) with higher porosity values reported, ranging between 12.6% and 18%, vary between 99 and 47 MPa. Vernik et al. [35] reported values of uniaxial compression tests obtained from sandstone samples collected on sedimentary basins with a worldwide distribution during hydrocarbon exploration. The sandstone samples with porosity values between 20% and 25% showed UCS (Co) of circa 70 and 50 MPa. On samples of unconsolidated conglomerates and conglomeratic sandstones collected from two wells drilled on oil and gas reservoirs at Alaska to about 3000 m up to 4000 m depth, Moos et al. [36] indicate UCS values about 35 to 83 MPa. 

Cnudde [37] describes the Bray sandstone as a Paleocene quartzitic sandstone with microcrystalline quartz cement. This author also mentions that it has been used as a building material since the Middle Ages on several constructions, including monuments in the city of Binche and in its vicinity. The Bray sandstone has an average grain size of 0.125–0.25 mm. Most of the grains are angular to sub-rounded. Quartz overgrowths prevail as silica cement type, and the higher porosity varieties show less cement. The average open porosity value is 13.7% (3.8–26.4%). The Mercury Intrusion Porosimetry data revealed that around 90% of the pores have a radius higher than 5 µm, and the radius mode obtained by µCT analysis is 19 µm on samples with open porosity values of 17.5–22.8%. The apparent density average value is 2281 kg/m^3^ (1900–2580 kg/m^3^). The average value of capillarity coefficient given by samples with open porosities between 17.5–22.5% is 400 g/m^2^s^1/2^, i.e., circa 24 kg/m^2^h^1/2^ (0.4 kg/m^2^s^1/2^). The test of water absorption under low pressure (Karsten tube) obtained on samples with 17% open porosity gave an average value of about 30 mL in 600 s, i.e., about 35 kg/m^2^h^1/2^. The previous value was obtained by the slopes of the linear regression of the initial part of the experimental curves. The average value of compressive strength given by samples with open porosity values of 20–22.5% is around 30 MPa. The average value of drilling resistance recorded in tests with a drilling rotation speed of 600 rpm and an advancing rate of 10 mm/min downward a drill hole of 5 mm of diameter, on samples with 17–26% open porosity, is about 22.5 N, i.e., 1.15 MPa. 

Hasníková and Zima [38] carried out absorption under low pressure tests and uniaxial compression tests on sandstone historical building stones. They used specimens of the following sandstones: one used for reconstructions of historical buildings in the Prague region, obtained nowadays from Hořice quarry and some blocks from Charles bridge; a coarse-grained sandstone (arkose) from Žehrovice; and a quartz sandstone with ferruginous cement and claystone fragments from the Petrín quarry. The average values of coefficient of water absorption under low pressure obtained by the Karsten pipe are given, varying from 14 to 557 kg/(m^2^ hod^0.5^) on Arkose, Hořice, and Petrín sandstones. The average compressive strength values of these sandstones obtained on cubic samples comprising a 50-mm-long edge range between circa 27 and 19 MPa. The compressive strength and the elastic modulus play a major role in the numerical simulation of the structural behavior of historical buildings and monuments. These mechanical parameters of rock reservoirs are also very important in the design and construction of oil and gas wells. 

The mechanical behavior of building stones under compression, which is dependent on their physical properties, is described by an analytical model [15]. A first step, characterized by experimental research on the physical and mechanical properties of stones under compression, is needed in order to define this model. The mechanical parameters (compressive strength and elastic modulus) allow the modeling of the compression curves using the stress–strain diagrams and a function dependent on physical properties, e.g., the porosity. However, the subsequent experimental procedures for determining the open porosity of rock samples based on Archimedes principle require several days or at least several hours if mercury intrusion porosimetry is performed. RILEM’s test of water absorption under low pressure on rock samples usually requires less than one hour if the open porosity values are higher than 15% or even just a few minutes if the samples with higher porosity values are tested [39]. The test of water absorption under low pressure is a true non-destructive test (NDT), whereas the porosity test procedures require the previous extraction of stone samples with adequate dimensions in order to respect monument integrity. 

The proposed method allows the gathering experimental data of the basic engineering properties of monument building stones and the use of non-destructive tests as a major advantage. This method is presented in the following sections, in which the test of water absorption under low pressure was used. Besides that, when the dependent function of the physical parameters is based on water absorption under low pressure, it is faster to obtain the simulation of the stress–strain diagrams than when it is based on the porosity, because the input data are more easily and more quickly obtained on more porous rocks by the former test than by the latter. In turn, this method can also be applied in the oil and gas industry, since the rocks of the best oil and gas reservoirs have higher values of porosity, and these small periods of testing can be an important laboratory time-saving procedure during the well construction activity. In the early stages of exploration, the proposed methodology can also be applied on rock samples showing a wide range of water absorption values, collected on outcrops used as analogs of near-surface or deep formations of oil and gas reservoirs. 

The compressive mechanical properties of building stones and materials of rock masses can be predicted by ultrasonic pulse velocity (UPV) and the rebound number of Schmidt hammer [40,41,42,43]. These properties are also used to estimate the porosity [42,44]. However, Selçuk and Nar [43] have found out that compressive strength is better estimated on rock specimens through the combined use of the ultrasonic pulse velocity Vp and the Schmidt hammer rebound number, because the affecting factors of the accuracy of the two combined techniques are minimized when the techniques are used together rather than separately. The water absorption under low pressure test is a simple and less expensive single test that allows the accurate simulation of the compression stress–strain diagrams. The authors of the present study did not find any related work in the literature with the simulation of stress–strain curves based on seismic velocity (V_P_) and/or the Schmidt rebound number.

The purpose of the present study consisted on the presentation of a new methodology based on a set of reliable procedures. This comprises the fast determination of a physical property such as water absorption under low pressure by use of non-destructive tests (NDT), beyond its traditional use, on each monument building stone, allowing its pre-peak compression stress-strain diagram to be obtained through previous calibration. Additionally, with this methodology the most important design parameters of loadbearing masonry can be predicted, such as the compressive strength, strain at failure, and even elastic modulus on the monuments’ blocks.

In order to accomplish the purpose referred, the following sections will present the selection of rock materials used in the study, the experimental methodology employed, and the test results regarding the physical properties, as well as the stress–strain curves of the sandstones. Then, based on these results, the various parameters were correlated, and an analytical model was developed to predict the stress–strain curves of the sandstones as a function of the coefficient of water absorption under low pressure.

## 2. Selection of Rock Materials 

**Figure 3:**
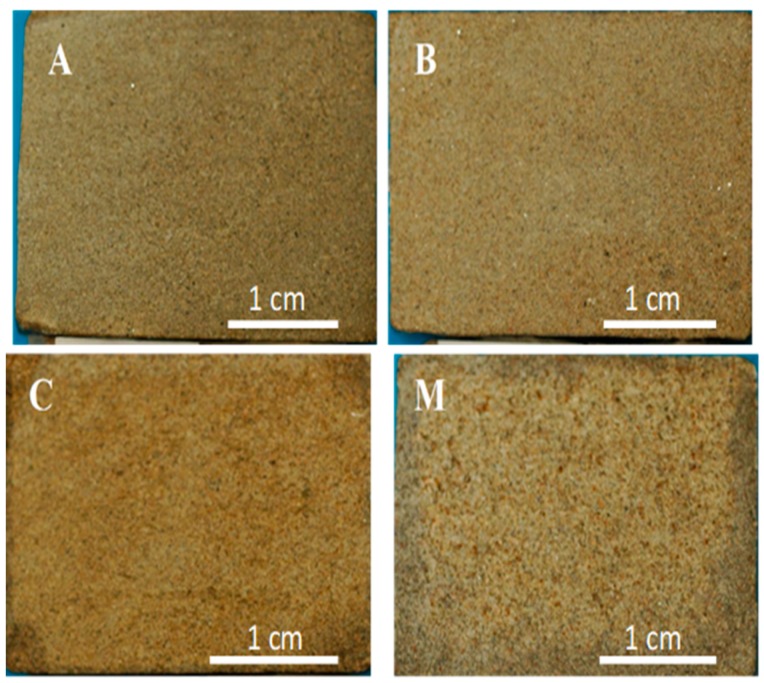
Hand specimen view of the four varieties of sandstone A, B, C, and M collected close to the monuments, identified on images. These varieties have average values of water absorption coefficient of, respectively: 0.8 kg/m^2^h^1/2^, 2.4 kg/m^2^h^1/2^, 6.2 kg/m^2^h^1/2^, and 26 kg/m^2^h^1/2^.

The two lithotypes that exist in the monuments include four varieties of sandstones that were identified in Atouguia da Baleia (Peniche) in the Portuguese Western region [1]. Walls of stone masonry were elected close to the built heritage, and some specimens similar to the stones in the monuments were extracted, as no coeval quarries or outcrops with similar materials were detected in areas close to Peniche. Physical tests were also carried out in order to obtain values of porosity and water absorption under low pressure of those samples collected, considering their resemblance based on surface appearance, mineralogical, texture, and structure properties. The representative samples of the four varieties in Figure 3 have similar average values of water absorption under low pressure coefficients compared to the stone in the monuments built in the Middle Ages (Figure 2): 0.8 kg/m^2^h^1/2^ (A), 2.4 kg/m^2^h^1/2^ (B), 6.2 kg/m^2^h^1/2^ (C), and 26 kg/m^2^h^1/2^ (M). These two lithotypes are designated as lithic arkose [1,15], according to Folk [44]. Varieties A and B included in lithotype A + B have about 30–32% quartz and 34–40% carbonates, and varieties C and M of the lithotype C + M have about 20–25% carbonates and 40–51% quartz. Both lithotypes were classified as lithic arkose with carbonate cement. Well-defined lineation is exhibited macroscopically in lithotype A + B. The A variety exhibited one major orientation of mica minerals, and the variety B showed no preferred orientations once the lineation was randomly distributed on thin sections under a polarizing microscope. Lineation was not identified in variety M, and thin sections of variety M showed two major orientations of mica minerals. The two lithotypes have around 4–6% of mica minerals.

Varieties A and B are usually fine-grained, and the average size of quartz and feldspar grains ranges from circa 0.1 to 0.13 mm. Varieties C + M are fine to medium-grained, and the average size of grains ranges between about 0.15 and 0.24 mm [1,15].

## 3. Experimental Methodology and Results

### 3.1. Introduction

Uniaxial compression tests were performed in the laboratory in order to obtain the stress–strain curves and the mechanical engineering parameters (compressive strength and elastic modulus) of the sandstone samples under study. Porosity and water absorption under low pressure tests were also carried out to obtain the sandstones’ physical properties. 

### 3.2. Preparation of Samples

The four varieties of sandstones were cut into prismatic samples of 50 × 50 × 100 mm^3^ and have a height-to-length ratio of two. Lithotype A + B showed macroscopic laminations and lineations, aligned parallel to the major axis. The prismatic specimens were randomly cut as no macroscopic lineations were found in the M variety sandstone [1].

### 3.3. Evaluation Tests on Physical Properties

**Figure 4:**
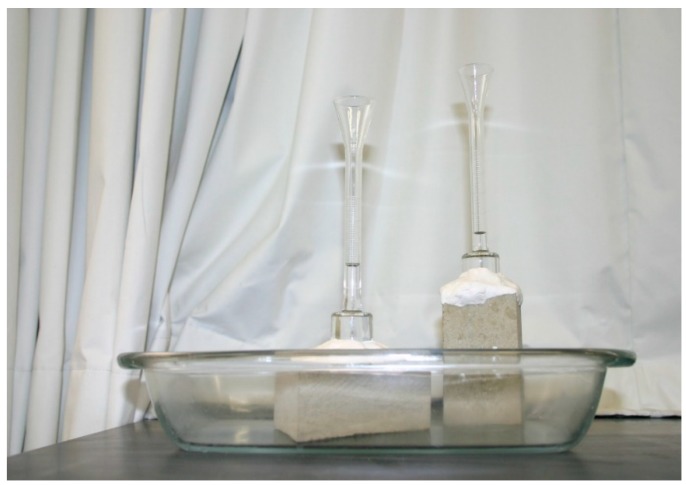
Testing equipment for the absorption of water under low pressure: Karsten glass pipe on sandstone samples inside a glass tray.

The values of porosity, density, and coefficient of water absorption under low pressure of the sandstones were given according to RILEM (1980) [45] and EN1936 (1999) [46] standards. A vacuum to saturate the samples is suggested by both standards. Archimedes-principle-based calculations were carried out to determine the pore volumes filled with water and allowed the attainment of porosity, bulk, and real densities. Other tests were carried out to obtain other physical properties, namely capillarity and mercury intrusion porosimetry [1]. The experimental apparatus is shown in Figure 4. The procedure of RILEM’s test of water absorption under low pressure [45] was carried out on sandstone specimens of prismatic shape. The Karsten tube was held on the sample surface by means of a replaceable and not harmful putty. The water filled the vertical pipe from the base upwards the top level ’’0’’. A total measuring height of 0.098 m and a volume of 4 cm^3^ were the main specifications of the pipe. The results were obtained from the water absorption under low pressure graphs (mass of fluid per area of its absorption) as a function of the square root of time, and the water absorption coefficient was given by the slope of a linear trend fitted to the first part of the test curve. The values of this parameter are usually given in the following sensitive units: kg/m^2^/√h.

All samples used in the mechanical characterization were subject to water absorption under low pressure tests in order to allow a direct correlation between the physical and mechanical parameters for water absorption. The experimental values of the coefficient of water absorption under low pressure (k) obtained for sandstones are shown in Table 3. 

The porosity values of A, B, C, and M varieties range between 3.6% and 18.6%. Concerning the values of the coefficient of water absorption under low pressure obtained on sandstones, there is a significant difference between varieties A (0.7 kg/m^2^h^1/2^) and M (31.8 kg/m^2^h^1/2^). The bulk density values of sandstones range between 2179 kg/m^3^ (variety M) and 2594 kg/m^3^ (variety A) [1]; this author presented pore size distribution of sandstone varieties B and M, obtained by mercury intrusion porosimetry (MIP). Microporosity, defined as the percentage of radii of voids smaller than 7.5 μm [47], is 80–85% in variety B and about 75% in variety M.

### 3.4. Monotonic Compression Tests 

The uniaxial compression tests carried out on the sandstones used a Seidner servo-controlled compression testing machine, model 3000D, from the laboratory of Structures of Nova University of Lisbon, Caparica, Portugal. The load capacity is up to 3000 kN and the piston stroke is 50 mm [1,15]. The tests were performed at a rate of 10 μm/s of axial displacement control carried out by the testing machine. Four diplacement transducers were used between plates, one per side and close to the sample. The resolution of these transducers of displacement is 100 × 10^−6^ strain/mm.

Recommendations from Fairhurst and Hudson, in the ISRM suggested methods [48] and ASTM D 7012 [49], allowed the calculation of the average axial strain of these sandstone specimens from the axial displacement of four LVDTs between plates. The upper and lower faces of the test specimens and the bearing faces of their corresponding plates were wiped clean as recommended. The complete details of the experimental procedures are given in Ref. [1,15]. The experimental values obtained for compressive strength (σ_c_) and strain at failure (ε_r_) of sandstones are shown on Table 3.

In uniaxial compression, information about the mechanical behavior of sandstones can be obtained through the stress–strain diagrams and follows five phases, as described in Ludovico-Marques et al. [15], citing Eberhardt et al. and Rocha [50,51]: (i) closure of existing cracks prior to compression; (ii) deformation under linear elastic regime; (iii) initiation of cracks and their stable growth; (iv) damage due to cracks and unstable growth of cracks; and (v) failure and post-peak zone of diagram.

Permeability continuously decreases through uniaxial compression evolution, covering all those stages up to failure. Permeability varies in rocks with higher porosity values as an effect of the applied stress paths (hydrostatic and triaxial compression, and uniaxial strain conditions/compression) following the same trend: Permeability continuously decreases as stress increases. Permeability increment only occurs under the triaxial compression stress path induced by tests carried out on rocks with lower porosity values, e.g., highly compacted sandstones, crystalline rock, and rocksalt [52,53,54,55,56]. This permeability increment is due to dilatant deformation under triaxial compression on brittle rocks. 

## 4. Modeling of Compression Behavior

### 4.1. Ludovico-Marques Global Model Used on Sandstones

As the physical properties play a major role in the compressive behavior of rocks, research efforts were undertaken for the use of water absorption data (Karsten pipe test) in order to obtain an adequate analytical model to carry out the description of the compressive behavior of sandstones. Ludovico-Marques [1] developed an analytical model to predict the compressive behavior of sandstones from their values of porosity, but in the present text, the model was adapted to carry out this prediction from the values of coefficient of water absorption under low pressure obtained by the Karsten pipe. The compressive stress, *σ*, on sandstones is calculated by Equation (1):

(1) $$\sigma =f\left(\varepsilon /\varepsilon_{R}\right)\times \sigma_{c}$$

where *σ_c_* is the compressive strength of rocks, and *f*(*ε/ε_R_*) is the shape function dependent on the strain *ε* normalized by the strain at failure (*ε*_R_). 

The shape function (*f*) is determined through the normalization of the compressive stress using the compressive strength, *σ_c_*, and is indicated as follows:

(2) $$f\left(\varepsilon /\varepsilon_{R}\right)=\frac{\sigma }{\sigma_{c}}$$

The results of the stress–strain curves of uniaxial compression tests on rocks allowed the calibration of the shape function. Ludovico-Marques [1], defined an analytical expression that accurately describes the pre-peak behavior, i.e., a cubic polynomial shape function in Equation (3), as follows:

(3) $$f\left(\frac{\varepsilon }{\varepsilon_{R}}\right)=-{\left(\frac{\varepsilon }{\varepsilon_{R}}\right)}^{3}+1.47x{\left(\frac{\varepsilon }{\varepsilon_{R}}\right)}^{2}+\frac{\left(\frac{\varepsilon }{\varepsilon_{R}}\right)}{2}$$

Ludovico-Marques et al. [15] reported that the shape function gives an output value of 1 when the strain has the value of *ε*_R_, and the coefficient 1.47 tends to 1.5.

The compressive stress, *σ*, given by Equation (4), considering the substitution of Equation (3) in Equation (1), is as follows:

(4) $$\sigma =\left[-{\left(\frac{\varepsilon }{\varepsilon_{R}}\right)}^{3}+1.47x{\left(\frac{\varepsilon }{\varepsilon_{R}}\right)}^{2}+\frac{\left(\frac{\varepsilon }{\varepsilon_{R}}\right)}{2}\right]\times \sigma_{c}$$

### 4.2. Correlations Between Compressive Strength, Strain at Failure, and Coefficient of Water Absorption Under Low Pressure 

**Figure 5:**
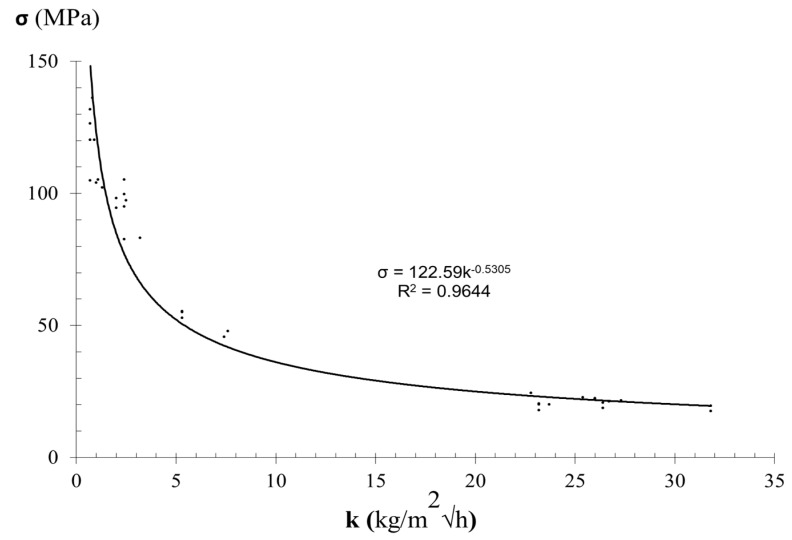
Relationship between compressive strength (σ_c_) and coefficient of water absorption under low pressure (k) obtained from sandstone samples.

Statistical correlations were obtained between experimental data of compressive strength, strain at failure, and the coefficient of water absorption under low pressure, which allowed the definition of the analytical model. The variation of the uniaxial compressive strength with the water absorption coefficient on the samples of sandstone is shown on Table 3 and Figure 5. Equation (5) is the regression that properly fits the experimental data reported in Table 3.

(5) $$\sigma_{c}=122.59k^{-0.5305}$$

The coefficient of determination (R^2^) of Equation (5) is 0.964. However, on less than 30% of sandstone specimens, the absolute differences between experimental and predicted compressive strength values, obtained through Equation (5), were higher than 15%.

**Figure 6:**
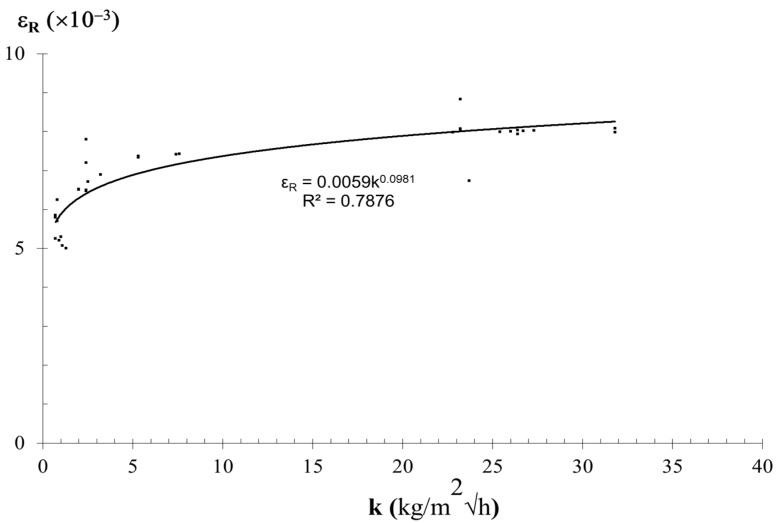
Variation between the strain at failure (ε_R_) and the water absorption (k) obtained on sandstone specimens.

The parameter coefficient of water absorption under low pressure also allows the description of the compression behavior of rocks as was earlier reported for porosity by Ludovico-Marques [1] andLudovico-Marques et al. [15]. A correlation between the strain at compressive strength and the physical properties of materials can be acomplished, assuring, as another important property, the complete description of the analytical model. The variation of strain at failure (ε_R_) with the coefficient of water absorption under low pressure on the samples of sandstone varieties are shown in Figure 6. As the coefficient of water absorption under low pressure obtained by the Karsten pipe decreases, the compressive strain at failure increases. This shows a clear trend between the variation of the compressive strain at failure and the variation of water absorption. 

The experimental data is properly fitted by the regression illustrated in Figure 6 and listed in Table 3 as Equation (6):

(6) $$\varepsilon_{R}=0.0059k^{0.0981}$$

The coefficient of determination (R^2^) of Equation (6) is 0.788.

The variation of values of strain at failure (ε_R_) obtained between each experimental data of A, B, C, and M sandstone varieties and their predicted data is shown on Table 4. Only around 10% of strain at failure (ε_R_) data of 40 specimens had absolute differences between the predicted and experimental values higher than 15%, and 20% of them differed by more than 10%; the average of the absolute difference of all samples was around 5%.

### 4.3. Analysis of Experimental and Predicted Results 

The analytical model was established by the replacement of the compressive strength and the strain at failure in Equation (4) shown above in the text. Thus, in Equation (4), the compressive strength (*σ_c_*) must be replaced by Equation (5), and the strain at compressive strength (*ε_R_*) by Equation (6). The following Equation (7) enables a prediction of the compressive strength and the definition of the relation between stress-strain curves and the water absorption under low-pressure for sandstones:

(7) $$\sigma =122.59k^{-0.5305}\;\{-{\left(\frac{\varepsilon }{0.0059k^{0.0981}}\right)}^{3}+1.47\;{\left(\frac{\varepsilon }{0.0059k^{0.0981}}\right)}^{2}+0.5\;\left(\frac{\varepsilon }{0.0059k^{0.0981}}\right)\}$$

Table 4 shows the values of strain at failure predicted by Equation (6) and the values of compressive strength determined by Equation (7), through the values of water absorption, reported in Table 3. The average value of absolute difference between the experimental and predicted data of the compressive strength of all samples was 10.5%. In 90% of the specimens of variety A and more than 60% of samples of variety B, the absolute difference between experimental and predicted values of compressive strength was lower than 15%, and was less than 25% in all of the latter. The values of compressive strength generally varied less than 15% between the experimental and derived data of sandstone samples of the C and M varieties.

**Figure 7:**
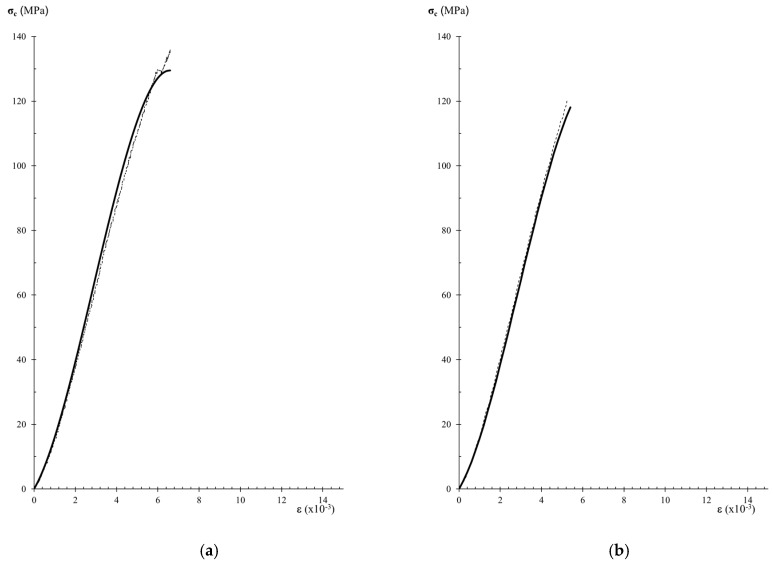

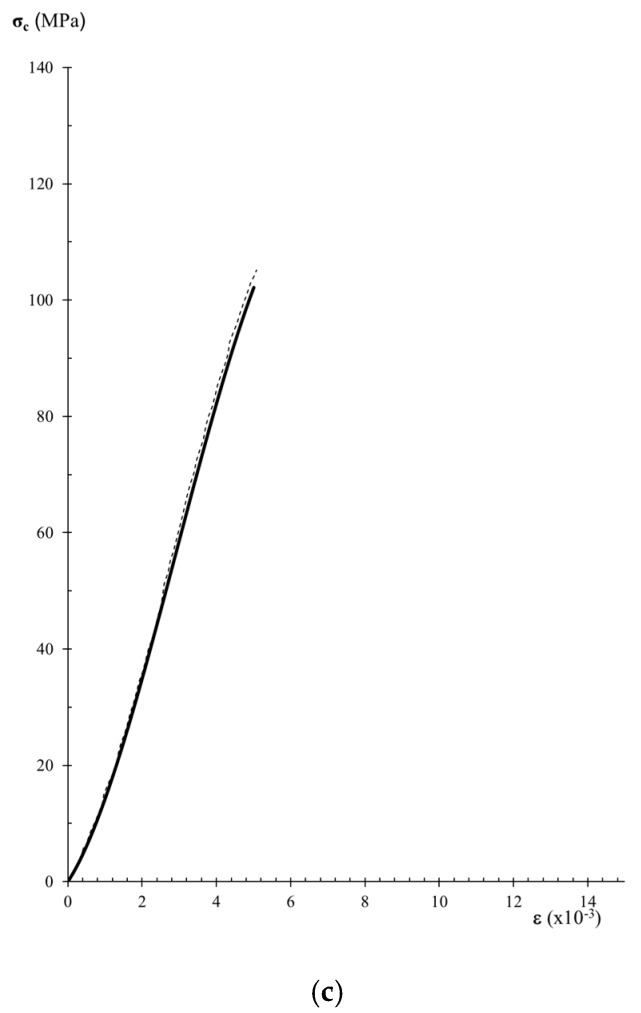
Analytical modeling of results of curves of variety A sandstone specimens. The dashed black curves are the stress-strain diagrams of experimental results, and the thicker black curves are the analytical stress–strain diagrams: (**a**) Specimen APN; (**b**) Specimen AP9; (**c**) Specimen AP5.

**Figure 8:**
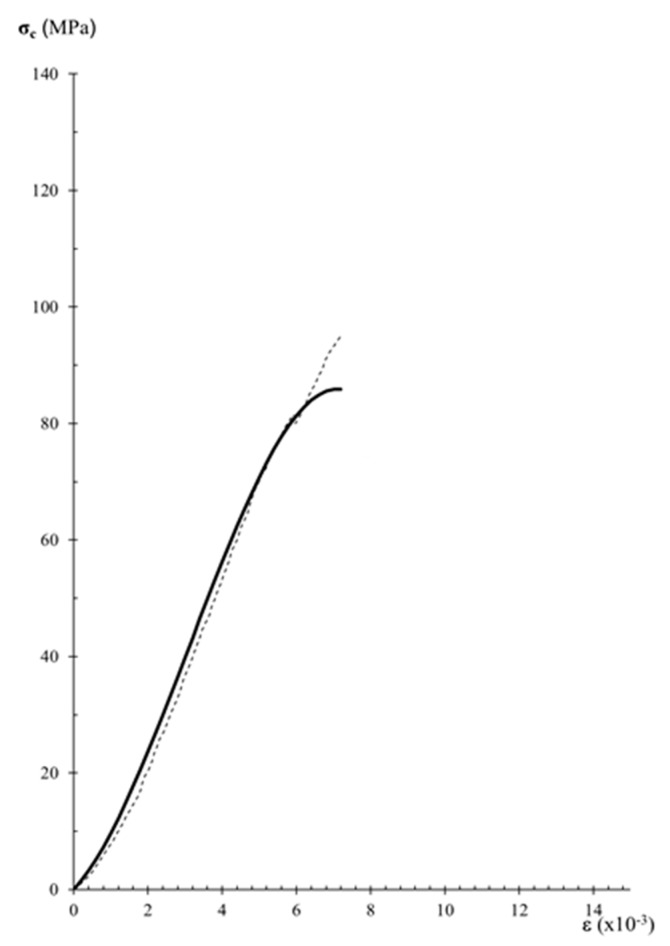
Analytical modeling of an experimental curve of variety B sandstone sample (BP3). The dashed black curve is the stress-strain diagram of experimental results and the thicker black curve is the analytical stress–strain-diagram.

**Figure 9:**
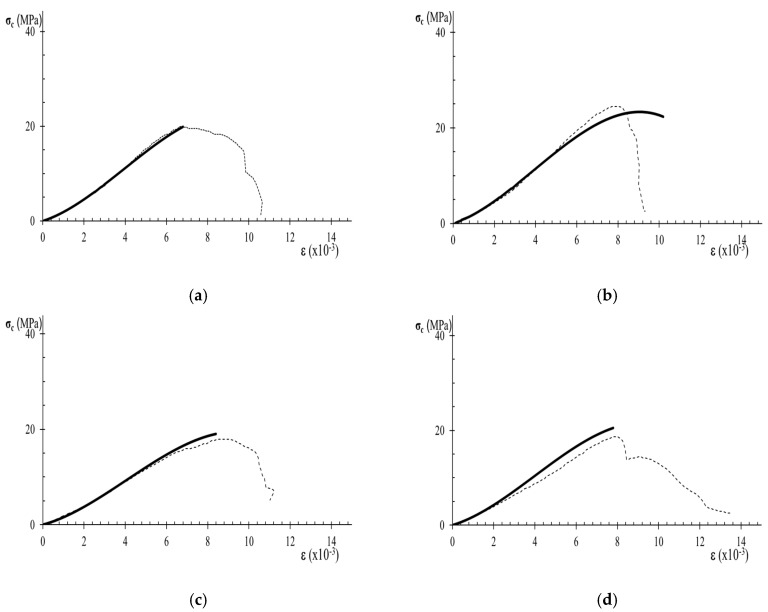
Analytical modeling of data from variety M sandstone specimens diagrams. The dashed black curves are the stress-strain diagrams of experimental results and the thicker black curves are the analytical stress–strain diagrams: (**a**) Specimen MP2; (**b**) Specimen MP3; (**c**) Specimen MP5; (**d**) Specimen MP1.

The comparison between experimental data of stress–strain curves and those obtained by the analytical expression of Equation (7) allows the assessment of the performance of the analytical model. Figure 7, Figure 8 and Figure 9, show experimental and analytical stress–strain curves. When comparing the curves in Figure 7, Figure 8 and Figure 9, a close fitting between the analytical and experimental results can be observed. The analytical expression is adequate for carrying out the description of the stress–strain behavior in the pre-peak hardening regime (Figure 7 and Figure 8). However, it can be observed that the analytical expression cannot predict the stress–strain behavior in the post-peak softening regime (Figure 9).

## 5. Conclusions

This paper provides experimental details of the behavior of sandstone under uniaxial compression. The stress–strain curves and the values of the major parameters that characterize the pre-peak behavior—compressive strength and strain at failure—were given. The physical properties of the sandstones, e.g., absorption of water under low pressure, greatly influences their compressive behavior, as shown by the experimental results. Higher values of the coefficient of absorption of water under low pressure of sandstone were clearly followed by smaller compressive strength values and higher values of strain at failure. An analytical model was derived in order to accomplish the description of the mechanical behavior of sandstones under compression. This model was developed from the general shape of the pre-peak stress–strain curves of the performed tests and an adequate definition of a cubic polynomial function. The strain normalization through the compressive stress, by using the compressive strength and the latter parameter, are the variables of the polynomial function, and the statistical correlations between these variables and the absorption of water under low pressure made it possible to state the final model. The evaluation of the performance of this analytical model was based on the comparison between the analytical stress–strain curves and the experimental stress–strain curves. The behavior under compression could be predicted when the absorption of water under low pressure was known because a good agreement was obtained between the analytical and experimental data of the pre-peak stress–strain curves. The water absorption under low pressure is a true NDT and a time-saving test for samples with higher porosities, and it allows the prediction of the mechanical behavior of the building sandstones and the sandstone reservoir rocks towards failure. However, the analytical model does not allow the prediction of the stress–strain behavior in the post-peak softening regime, which will be the scope for further development.

## Figures and Tables

**Table 1:** Mineralogical characterization of building sandstones of world cultural heritage.

Countries		Tunisia	Egypt	Jordan		Switzerland			Germany	Italy	Cambodja		
	Origin	Aqueduct (Zoghlami et al., 2004)	Gebel Silsila (Fitzner et al., 2003)	Petra tombs (Heinrichs, 2005)		Fribourg (“Suisse plateau”) (Félix, 1977)			Birkenfeld Monastery (Tiano et al., 2000)	Strozzi Palace (Malesani and Vannucci, 1974) (Banchelli et al., 1997)	Angkor (Uchida et al., 1999)		
	Lithology	Medium to fine grained sandstones	Fine grained sandstones (white to yellow brown)	Medium to fine grained sandstones (several colors, including white)		Blue molasse	Yellow molasse	Villarlod sandstone	Sandstones with rock fragments (green to gray)	Lithic arenites with carbonate cement (Pietraforte)	Grey yellow sandstone	Red sandstone	Green grauwacke
	Stratigraphy	Miocene Fortuna Formation.	Cretaceous Qoseir Formation.	Cambrian Umm Ishrin, Formation	Ordovician Disi Formation.	Extra Alpin sea molasse.			Triassic, middle Keuper (Schilfsandstein)	Upper Cretaceous (External Ligurides)	-	-	-
Composition (%)	Quartz	69–84	65–90	Matrix-rich sandstone	quartz. sandstone	60–70			89	Major	+	++	+
	Feldspars	0–1	0–8			10–15			9	Major k-feldspar and plagioclases	+		+
	Micas		0–0.5			+			1		+	-	+
	Clays	0–10	0–10; *25			-			++	Illite and chlorite-vermiculite	-	-	-
	Calcite		0–3			20–30			-	Major calcite and dolomite	-	-	-
	others		1–5			3–5 (glauconite)			1 (chlorite)	chlorite	+ (goeth.)	+ (hemat., goeth)	-
Grain size analysis	Average size (mm)	0.15–0.42	0.1–0.2	0.17	0.31	0.4–0.5	0.3–0.4	0.1–0.2 0.2–0.3	0.3 (0.1–0.5)	0.08 (max. 0.25)	0.2–0.3	0.1–-0.2	-
	Distribution	Slight to poor graded	Slight to poor graded	-	-	graded			Slight graded	Slight graded	Slight graded	Poor graded	graded

* Also was found; + contain; ++ contain very.

**Table 2:** Physical and mechanical properties of building sandstones of world cultural heritage.

Origin	Aqueduct (Zoghlami et al., 2004)	Gebel Silsila (Fitzner et al., 2003)	Petra Tombs (Heinrichs, 2005)		Fribourg (“Suisse Plateau”) (Félix, 1977)			Birkenfeld Monastery (Tiano et al., 2000)	Strozzi Palace (Malesani and Vannucci, 1974) (Sorace, 1996)	Angkor (Uchida et al., 1999)		
Median pore radius (µm)	20–50 (Mode)	75–110 10 clayey sandstone	13	115	-	-	-	100–200 (average)	-	-	-	-
Unimodal/multimodal	Unimodal	Unimodal	-	-	-	-	-	-	-	-	-	-
Porosity (%)	17.5–26; 23 (open)	25–35	17.4	21.3	15	18.2	14.9	19.2 ± 0.7 (open)	1.8–5.5 (open)	13–19 (open)	11–15 (open)	2 (open)
Densities: bulk ^1^, real ^2^ (kg/m^3^)	1950–2150 ^1^ 2550–2600 ^2^	1800–2000 ^1^ 2600–2750 ^2^	-	-	2260–2280 ^1^ 2670 ^2^	2180–2190 ^1^ 2660–2670 ^2^	2240–2270 ^1^ 2640–2660 ^2^	2160 ± 20 ^1^ 2670 ± 10 ^2^	2580–2610 ^2^	2100–2400 ^1^	2100–2400 ^1^	2600–2700 ^1^
Maximum water absorption (%)	11 ± 1.7	-		-	-	-	-	-	1–2.5	-	-	-
Water absorption coefficient/ /capillarity (kg/m^2^h^1/2^) or permeability (mD)	3.1 ± 1.6 (0.51 ± 0.27 kg/m^2^s^1/2^)	-	-	-	3.2–3.4 (┴) 3.3–3.5 (║)	5.3–5.4 (┴) 6.2–6.5 (║)	2.2–2.4 (┴) 3.6–3.7 (║)	-	< 1 mD	-	-	-
Expansion (mm/m)	None	-	-	-	1.63–1.74 (┴) 1.58–1.68 (║)	2.5–2.76 (┴) 1.71–2.10 (║)	2.27–2.28 (┴) 1.35–1.44 (║)	-	-	-	-	-
Compressive strength (MPa)	15.6 ± 7.9	-	-	-	46.3–55.4 (┴) 46.7–51.7 (║)	30.6–40.4 (┴)	62.4–71.0 (┴) 51.6–57.3 (║)	52.3 ± 10.9	121.2-140	32–44 estimated	43 estimated	80 estimated
Dynamic elastic modulus (GPa)	-	-	-	-	7.2–8.4 (║)	17.5–18.1 (║)	10.3–12.1 (║)	-	38.3	-	-	-
Bending strength (MPa)	-	-	-	-	2.55–2.70 (┴)	2.49–3.65 (┴)	9.60–9.90 (┴)	-	9.4	-	-	-
Tensile strength (MPa)	-	-	-	-	1.17–1.28 (║)	0.6–0.7 (║)	2.24–2.74 (║)	-	3.9	-	-	-
UPV of P waves (km/s)	-	1.2–2.2 (┴) 1.3–2.6 (║)	-	-	1.7 (┴) 1.9 (║)	1.2–1.4 (┴) 1.7–1.8 (║)	1.7–2.1 (┴) 2.3–2.5 (║)	-	-	1.9–3.2 (║)	3.9–4.0 (║)	4.4 (║)
Rebound number	-	-	-	-	45 (┴)	31 (┴) & 29 (║)	47 (┴) & 42 (║)	-	-	45–54	53	64
Drilling strength	-	0.5–1.2 (┴)	4.5	2.0	-	-	-	15 N	-	-	-	-

**Table 3:** Values of physical and mechanical properties of sandstones.

Variety	Specimens	σ_c_ (MPa)	ε_r_ (×10^−3^)	k (kg/m^2^/√h)
A	AP38	126.4	5.7900	0.7
	AP39	131.8	5.7931	0.7
	AP53	148.2	5.6968	0.8
	AP96	104.9	5.8542	0.7
	AP1	102.3	5.0000	1.3
	AP5	105.2	5.1000	1.1
	AP6	104.0	5.3000	1.0
	AP9	120.3	5.2000	0.9
	AP11 (N)	136.2	6.2500	0.8
	AP13 (X)	135.7	6.6300	0.9
B	BP6	99.7	6.4725	2.4
	BP27	82.6	6.4932	2.4
	BP32	83.1	6.8965	3.2
	BP45	97.3	6.7059	2.5
	BP72	98.2	6.5136	2.0
	BP3	95.0	7.2000	2.4
	BP13	105.3	7.8000	2.4
	BP	94.5	6.5136	2.0
C	CP18	47.8	7.4265	7.6
	CP24	45.7	7.4139	7.4
	CP50	52.8	7.3366	5.3
	CP40	55.1	7.3497	5.3
	CP87	55.3	7.3627	5.3
M	MP12	20.0	8.0615	23.2
	MP13	20.4	8.0708	23.2
	MP9	22.0	8.0048	26.0
	MP10	22.7	7.9953	25.4
	MP11	20.7	8.0333	26.4
	MP12M	22.3	8.0048	26.0
	MP92	21.3	8.0144	26.7
	MP109	19.6	8.0800	31.8
	MP110	22.4	8.0048	26.0
	MP111	21.6	8.0239	27.3
	MP112	21.3	8.0144	26.7
	MP113	22.2	8.0048	26.0
	MP1	18.7	7.9000	26.4
	MP2	20.0	6.7300	23.7
	MP3	24.5	7.9800	22.8
	MP5	17.9	8.8300	23.2
	MP6	17.6	7.9800	31.8

**Table 4:** Values of mechanical data and mechanical properties of sandstones predicted.

Variety	Specimens	σ_c_ (MPa)	ε_r_ (×10^−3^)	Predicted ε_r_ (×10^−3^)	Predicted and Experimental Absolute Difference ε_r_ (%)	Predicted σ_c_ (MPa)	Predicted and Experimental Absolute Difference σ_c_ (%)
A	AP38	126.4	5.7900	5.6971	1.6	146.0	15.5
	AP39	131.8	5.7931	5.6971	1.7	146.1	10.9
	AP53	148.2	5.6968	5.7723	1.3	132.1	10.9
	AP96	104.9	5.8542	5.6971	2.7	147.6	40.7
	AP1	102.3	5.0000	6.0538	21.1	85.5	16.5
	AP5	105.2	5.1000	5.9554	16.8	96.8	8.0
	AP6	104.0	5.3000	5.9000	11.3	106.8	2.7
	AP9	120.3	5.2000	5.8393	12.3	112.0	6.9
	AP11 (N)	136.2	6.2500	5.7723	7.6	144.9	6.4
	AP13 (X)	135.7	6.6300	5.8393	11.9	142.8	5.2
B	BP6	99.7	6.4725	6.4291	0.7	75.2	24.5
	BP27	82.6	6.4932	6.4291	1.0	75.5	8.6
	BP32	83.1	6.8965	6.6131	4.1	66.9	19.5
	BP45	97.3	6.7059	6.4549	3.7	76.0	21.9
	BP72	98.2	6.5136	6.3151	3.0	84.9	13.5
	BP3	95.0	7.2000	6.4291	10.7	83.7	11.9
	BP13	105.3	7.8000	6.4291	17.6	90.7	13.9
	BP	94.5	6.5136	6.3151	3.0	84.9	10.1
C	CP18	47.8	7.4265	7.1988	3.1	41.8	12.5
	CP24	45.7	7.4139	7.1800	3.2	42.5	7.1
	CP50	52.8	7.3366	6.9487	5.3	51.8	1.8
	CP40	55.1	7.3497	6.9487	5.5	51.9	5.8
	CP87	55.3	7.3627	6.9487	5.6	52.0	5.9
M	MP12	20.0	8.0615	8.0317	0.4	22.5	12.6
	MP13	20.4	8.0708	8.0317	0.5	22.5	10.5
	MP9	22.0	8.0048	8.1220	1.5	20.8	5.4
	MP10	22.7	7.9953	8.1034	1.4	21.1	7.1
	MP11	20.7	8.0333	8.1341	1.3	20.7	0.1
	MP12M	22.3	8.0048	8.1220	1.5	20.8	6.7
	MP92	21.3	8.0144	8.1432	1.6	20.5	3.8
	MP109	19.6	8.0800	8.2840	2.5	18.5	5.6
	MP110	22.4	8.0048	8.1220	1.5	20.8	7.1
	MP111	21.6	8.0239	8.1609	1.7	20.2	6.3
	MP112	21.3	8.0144	8.1432	1.6	20.5	3.8
	MP113	22.2	8.0048	8.1220	1.5	20.8	6.3
	MP1	18.7	7.9000	8.1341	3.0	20.3	8.8
	MP2	20.0	6.7300	8.0485	19.6	18.5	7.3
	MP3	24.5	7.9800	8.0180	0.5	22.5	8.0
	MP5	17.9	8.8300	8.0317	9.0	24.7	37.8
	MP6	17.6	7.9800	8.2840	3.8	18.3	3.9
